# Icariin-Loaded Milk-Derived Extracellular Vesicles: Protective Effect on Inflammatory Bone Defects via HIF-1α

**DOI:** 10.3390/pharmaceutics18070797

**Published:** 2026-06-29

**Authors:** Ming Dong, Xinxin Yu, Shuo Liu, Yue Han, Wenqing Han, Lina Wang, Weidong Niu

**Affiliations:** School of Stomatology, Dalian Medical University, Dalian 116044, China

**Keywords:** icariin, small extracellular vesicles, inflammatory, bone defects

## Abstract

**Objective:** Icariin (ICA) is an active small molecule extracted from Epimedium, possessing therapeutic potential for inflammatory bone destruction. Small extracellular vesicles (MEVs) derived from bovine milk are safe and efficient drug delivery carriers. We aimed to explore the potential of ICA-loaded bovine milk EVs (ICA-MEVs) to repair inflammatory bone defects in an inflammatory microenvironment and investigated the underlying molecular mechanism, providing new ideas for the treatment of inflammatory bone defects. **Methods:** We fabricated icariin (ICA)-loaded milk-derived extracellular vesicles (ICA-MEVs) embedded in GelMA hydrogel and systematically evaluated the in vivo repairing efficacy against lipopolysaccharide (LPS)-induced inflammatory calvarial bone defects via micro-CT, HE staining, Masson staining and immunohistochemistry. Subsequent in vitro cellular experiments were carried out to uncover the regulatory mechanism by which ICA-MEVs promotes LPS-inhibited osteoblast proliferation and osteogenic differentiation. **Results**: ICA-MEVs significantly promoted the repair of inflammatory bone defects, upregulated osteogenic factors such as BMP-2, OCN, and Runx-2, and reduced the levels of IL-1β and TNF-α. ICA-loaded MEVs facilitated the proliferation and osteogenic differentiation of MC3T3-E1 osteoblasts while alleviating cellular inflammatory activation. Mechanistically, ICA-MEVs promoted bone repair by elevating LIM1 expression. Elevated LIM1 bound to the endogenous HIF-1α promoter and triggered subsequent transcriptional activation of HIF-1α. **Conclusions:** Under inflammatory conditions, ICA-MEVs effectively promoted the proliferation and differentiation of MC3T3-E1 cells and inhibited the expression of inflammatory factors. Mechanistically, ICA-MEVs upregulated HIF-1α transcription and expression by potentiating the LIM1-mediated transcriptional activation of the HIF-1α promoter, thereby facilitating inflammatory bone repair. Although milk-derived EVs exhibited favorable safety profiles in this preclinical study, comprehensive detection of immunogenicity and long-term adverse reactions will be necessary in follow-up research to support clinical transformation.

## 1. Introduction

Alveolar bone resorption and jawbone defects induced by inflammation are among the most common clinical manifestations of various oral diseases [1]. Epimedium, a traditional Chinese herbal medicine with a history spanning hundreds of years, contains icariin (ICA), a typical flavonoid compound that exhibits excellent antioxidant, anti-inflammatory, antibacterial, anti-apoptotic, and osteoblast differentiation- and mineralization-promoting activities [2]. However, its poor solubility and low bioavailability have hindered its widespread clinical application. Therefore, researchers have explored the use of drug delivery carriers to enhance the bioavailability of ICA. Negrescu et al. prepared ICA-loaded titanium dioxide nanotubes and found that this material increased the thickness of new bone tissue; however, its preparation process is complex and time-consuming. Other studies have adopted tissue engineering technology to develop materials for bone defect repair [3]. Zhou et al. co-cultured ICA-loaded polyethylene glycol-co-caprolactone (PGCL) porous microcarriers with bone marrow mesenchymal stem cells, and in vivo experiments revealed that this complex exerted an excellent synergistic effect in repairing rat skull defects [4]. Naked EVs undergo rapid clearance via body fluid circulation and immune phagocytosis, leading to brief retention at bone defects. Hydrogel or scaffold encapsulation physically anchors EVs to avoid off-target loss and enables gradual, long-term release matching bone remodeling cycles. Sustained local EV levels maintain stable pro-osteogenic, anti-inflammatory and angiogenic activity; together with the biomaterials’ 3D supportive microenvironment for cell growth, this synergistically improves bone regeneration versus free EV administration [5,6].

LPS-induced inflammatory bone lesions inhibit osteogenesis and bone repair. Icariin has anti-inflammatory and osteogenic effects but suffers from unstable local retention. With biocompatible and sustained-release properties, MEVs can serve as ideal carriers, and GelMA hydrogel can achieve in situ immobilization of bioactive substances. This study fabricated an MEV-ICA composite hydrogel and explored its therapeutic effects and mechanisms against inflammatory bone defects via in vitro osteoblast experiments and in vivo mouse skull defect models. C57BL/6 mice share high homology with humans in bone metabolism and inflammatory signaling pathways. The LPS-stimulated mouse critical-sized calvarial defect model can mimic the pathological characteristics of human inflammatory bone injury and thus is suitable for evaluating biomaterial-mediated bone repair. This research provides valuable theoretical and translational references for clinical treatment of inflammatory bone defects.

## 2. Materials and Methods

### 2.1. Isolation Milk-Derived Extracellular Vesicles (MEVs)

Multiple orthogonal detection methods were applied to identify MEV features and sample purity in strict accordance with MISEV2023 reporting standards [7]. A GEM-2000EX transmission electron microscope (TEM, EOL Ltd., Tokyo, Japan) was used to observe vesicle ultrastructure, while particle size distribution and vesicle concentration were measured using a ZetaView^®^ BASIC nanoparticle tracking analysis (NTA, Particle Metrix GmbH, Meerbusch, Germany) platform. Immunoblotting was performed to test universal extracellular vesicle biomarkers CD81, CD63, CD40 and ALIX, with all primary antibodies diluted at a ratio of 1:1000.

TEM images displayed typical cup-shaped vesicle structures with little residual cell debris. NTA results presented a unimodal particle distribution ranging from 30 to 150 nm, which excluded excessively large particulate contaminants. The obvious positive signals of CD63, CD81 and ALIX further eliminated interference from soluble protein impurities, as non-vesicular components rarely carry multiple endosome-originated vesicle proteins. Collectively, these multi-index results demonstrated the satisfactory purity of our extracted MEVs. It should be noted that extra quantitative assays for purity evaluation were not arranged in this research, which we acknowledge as an experimental deficiency.

### 2.2. Drug Encapsulation

A total of 0.001 g ICA (Chengdu Must Biotechnology Co., Ltd., Chengdu, China) powder was dissolved in 1 mL DMSO to prepare 1 mg/mL. ICA was loaded into the MEVs via 24 h incubation at 22 °C at a BCA-calibrated 1:9 mass ratio, which was optimized from gradient screening. The final DMSO concentration was controlled under 0.1% (*v*/*v*) to eliminate solvent interference.

Unbound ICA was removed by centrifugation (10,000× *g*, 10 min). ICA-MEVs were harvested via ultracentrifugation (135,000× *g*, 2 h), resuspended in PBS and stored at −80 °C. Loading efficiency was determined by spectrophotometry and HPLC with MEV protein normalization.

UV spectrophotometry preliminarily determined ICA loading efficiency. Gradient ICA standards were detected at the characteristic UV wavelength to generate a calibration curve. After ultracentrifugation to remove free ICA, resuspended ICA-MEV was assayed identically, with loading efficiency calculated as: loading efficiency (%) = (mass of encapsulated ICA/total MEV mass) × 100. Precise ICA quantification was performed via HPLC-MS/MS. ICA-MEV suspensions were mixed with acetonitrile (1:5, *v*/*v*), incubated at room temperature for 2 min and centrifuged at 1000× *g* for 10 min to collect supernatants. Matched ICA standards underwent parallel pretreatment for calibration, and all samples were diluted 20-fold with ddH_2_O before detection. Analytes were separated on an Agilent 1200 HPLC coupled to an API 3200 triple quadrupole mass spectrometer equipped with a ZORBAX SB-C18 column (4.6 mm × 150 mm, 5 μm) at ambient temperature. Binary eluents of 0.1% aqueous methanol and acetonitrile (65:35, *v*/*v*) flowed at 0.5 mL/min. Encapsulated ICA content and loading efficiency were quantified via calibration curves based on target peak areas.

### 2.3. Cell Culture

MC3T3-E1 osteoblast cells were obtained from Wuhan Pricella Biotechnology Co., Ltd. (Wuhan, China). The cells were cultured in standard DMEM medium containing 10% fetal bovine serum (FBS; Aojing Biotechnology, Weifang, China), 100 U/mL penicillin and 100 μg/mL streptomycin. Cell incubation was performed at 37 °C in a humidified atmosphere with 5% CO_2_ for routine growth and maintenance.

### 2.4. Preparation of GelMA Hydrogel Composites

Twenty micrograms GelMA powder (EFL, Suzhou, China) was mixed with 100 μL photoinitiator, heated at 60 °C in the dark and shaken every 6 min, repeated three times. PBS, miR-29b, MEVs or MEVs/miR-29b were then added at a 1:1 ratio. After mixing, the mixture was molded into 3 mm sheets, photocured and stored at 4 °C. ICA powder, lyophilized MEVs or ICA-MEVs were separately resuspended in 40 μL PBS (4.25 μg/μL), while the PBS group received an equal volume of blank PBS. Each suspension was mixed thoroughly with 500 μL GelMA precursor solution. The mixed solution was divided into six equal aliquots in 6-well plates and crosslinked rapidly under LED light to form hydrogel sheets.

### 2.5. Establishment of a Mouse Skull Defect Model

All animal procedures were approved by the Institutional Animal Care and Use Committee of Dalian Medical University (Approval No. AEE23006). Experimental mice were provided by Dalian Medical University and housed in a standard environment with a 12 h light/dark cycle at 22 ± 2 °C.

To establish an inflammatory cranial defect model, eight 8-week-old male C57BL/6 mice were randomized into control and LPS groups (*n* = 4). After intraperitoneal anesthesia with 30 mg/kg sodium pentobarbital, a 1.5 × 1.5 mm critical-sized calvarial defect was created via dental drill. The LPS group received seven local injections of 200 μg LPS every 48 h, whereas the control group received no treatment. Cranial tissues were collected 2 weeks later, and successful model construction was verified by macroscopic observation, micro-CT, HE, Masson staining and IHC.

For treatment experiments, twenty-four 8-week-old male C57BL/6 mice were randomly assigned to five groups (*n* = 6). Four GelMA-based formulations containing PBS, free ICA, blank MEVs or ICA-MEVs were implanted into cranial defects, with corresponding dosages of 400 μg ICA and 3200 μg for both MEV groups. Mice were randomly grouped, and all micro-CT and histological assessments were performed in a blinded manner by two researchers. All mice were euthanized 2 weeks after implantation, and cranial samples were harvested for subsequent analysis.

### 2.6. Western Blotting

Cellular total protein was extracted using RIPA lysis buffer (Kage Biotechnology Co., Ltd., Shanghai, China). Membranes were probed overnight at 4 °C with primary antibodies, including HIF-1α (1:1000), LIM1 (1:500), RUNX2 (1:500), ALP (1:1000) and GAPDH (1:5000) from Huabio (Hangzhou, China), as well as CD86 (1:1000), CD206 (1:1000) and Arg-1 (1:1000) from ABclonal (Woburn, MA, USA). Following incubation with HRP-conjugated secondary antibodies (1:5000; ABclonal, Woburn, MA, USA) for 1 h at room temperature, target protein bands were visualized via ECL chemiluminescent substrate and imaged using a Bio-Rad gel imaging system (Bio-Rad, Hercules, CA, USA) for protein expression detection.

### 2.7. Quantitative Reverse Transcription Polymerase Chain Reaction

Total cellular RNA was harvested with the UNIQ-10 Column Trizol Total RNA Extraction Kit (Sangon Biotech, Shanghai, China). Purified RNA was then reverse-transcribed into complementary DNA (cDNA) using a commercial Reverse Transcription Kit (Aikangde Biotechnology, Shenzhen, China) in strict accordance with the manufacturer’s standard protocols.

### 2.8. Transcriptome Sequencing and Analysis

Transcriptomic profiling was outsourced to Novogene Co., Ltd. (Shanghai, China). Sequencing libraries were built for the Illumina platform using the NEBNext Ultra™ Kit (New England Biolabs, Ipswich, MA, USA), with 1 μg purified RNA per sample and specific barcode indices applied for sample discrimination. All library preparation workflows strictly followed official protocols, and three biological replicates were included in each group to ensure data robustness. Following standard data normalization, multivariate statistical analyses, including principal component analysis and hierarchical clustering, revealed high similarity within groups and apparent transcriptional divergence between groups, indicating stable sample quality and valid group division. Genes with significant expression differences were identified with unified criteria: |log2(fold change)| ≥ 1 and adjusted *p*-value < 0.05. The Benjamini–Hochberg approach was employed for FDR correction to eliminate false positives induced by multiple statistical tests. Qualified differential genes were retained for subsequent functional enrichment exploration. All raw sequencing datasets were uploaded to a public repository for open sharing.

### 2.9. Immunohistochemistry (IHC)

Collected tissue specimens were paraffin-embedded and cut into continuous 3 μm-thick sections. All prepared tissue slices and matched experimental reagents were loaded into an automatic immunohistochemical staining machine for standardized staining. Primary antibodies adopted in this study included anti-BMP2 (1:200, Huabio), anti-OCN (1:500, Abcam, Cambridge, UK), anti-Runx-2 (1:200, Huabio), anti-IL-1β (1:200, Huabio) and anti-TNF-α (1:200, Huabio). Immunohistochemical staining images were acquired via a Nikon microscope equipped with a CCD image sensor. To ensure consistent imaging conditions, all sections were photographed at a fixed magnification of 400× in a unified light environment with identical aperture and light source parameters. Five random visual fields were captured for each tissue section. The positive expression levels of target proteins were quantitatively analyzed using Image-Pro Plus 6.0 software (Media Cybernetics, Rockville, MD, USA).

### 2.10. Statistical Analysis

All experimental data were expressed as the mean ± standard deviation (SD). Statistical analyses were carried out with IBM SPSS 13.0 software (IBM Corp., Armonk, NY, USA). Two-tailed Student’s *t*-test and one-way ANOVA were applied for intergroup comparisons, and a *p* value below 0.05 was defined as statistically significant. All statistical procedures followed standardized analytical specifications. Distinct sample replicates were set according to experimental types, including six biological replicates for animal studies, five for cell experiments and four for tissue tests. In addition, all detections were performed technically in triplicate to minimize operational errors, and averaged values were used for subsequent analysis to prevent pseudo-replication. The sample size of each group was pre-estimated through statistical power analysis with a power of 0.8 and α value of 0.05. Multiple comparison correction was conducted to reduce false positive outcomes, thereby guaranteeing the reliability and repeatability of final statistical results.

## 3. Results

### 3.1. Identification of MEVs and ICA-MEVs

**Figure 1:**
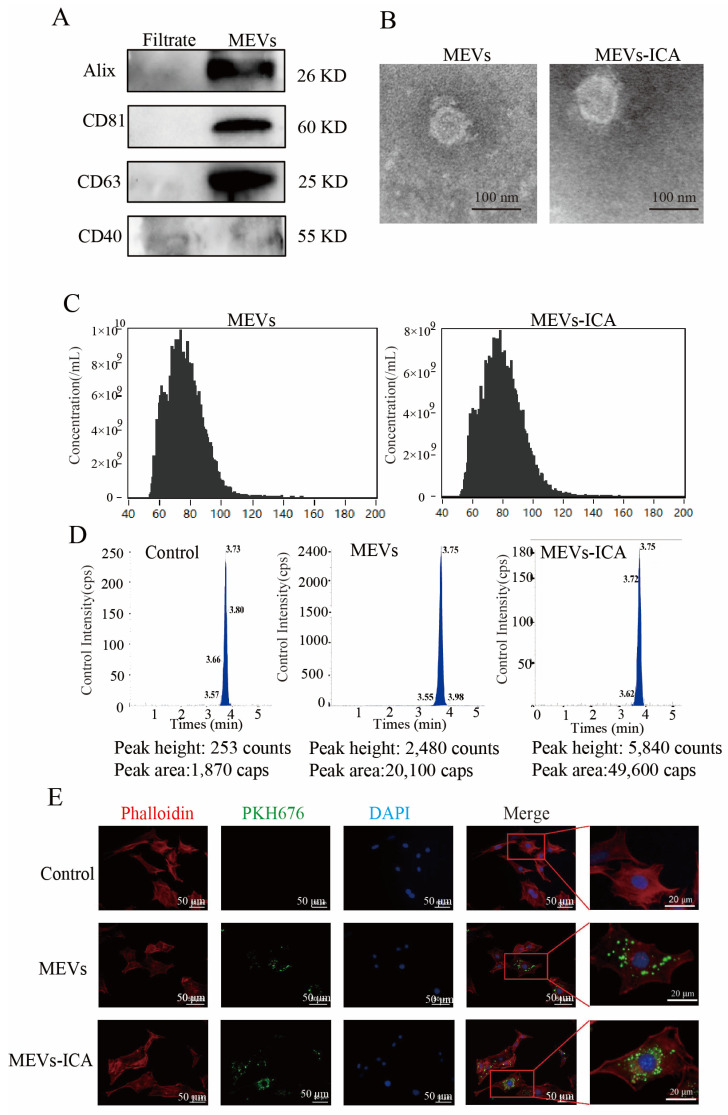
Characterization of MEVs and ICA-MEVs. (**A**) Western blotting detected the surface marker expression of MEVs. (**B**) TEM displayed MEV morphology (scale bar = 100 nm). (**C**) NTA analyzed the particle size distribution of MEVs. (**D**) HPLC verified ICA loading in ICA-MEVs. (**E**) Cellular uptake of MEVs and ICA-MEVs was observed in MC3T3-E1 cells.

Western blotting analysis demonstrated that MEVs were positively enriched for the exosomal surface markers CD81, Alix, and CD63, while showing low expression of the microvesicle marker CD40 (Figure 1A). TEM revealed that both MEVs and ICA-MEVs displayed a characteristic round or oval morphology with a distinct lipid bilayer structure (Figure 1B). Nanoparticle tracking analysis (NTA) was utilized to characterize the particle diameter of prepared vesicles. The average particle size of blank MEVs was determined to be 77.1 nm, while ICA-MEVs exhibited a slightly larger average size of 81.3 nm. Both vesicle formulations possessed particle sizes consistent with the standard dimensional range of extracellular vesicles, confirming successful vesicle preparation (Figure 1C). To verify cellular internalization, MC3T3-E1 cells were incubated with PKH67-labeled MEVs and ICA-MEVs for 24 h. Fluorescence imaging showed green signals from PKH67-labeled vesicles, blue nuclear staining by DAPI, and red labeling of the cytoskeleton by phalloidin. HPLC quantification revealed a 5–8% ICA loading efficiency for MEV-ICA (Figure 1D), corresponding to 5–8 μg encapsulated ICA per 100 μg MEV total protein (0.05–0.08 μg ICA per 1 μg MEV protein) (Figure 1D). The fluorescent signals were predominantly distributed in the perinuclear cytoplasm, confirming that MC3T3-E1 cells could effectively take up milk-derived MEVs and ICA-MEVs under inflammatory conditions (Figure 1E).

### 3.2. ICA-MEVs Promoted Bone Repair in a Mouse Model of LPS-Induced Skull Inflammatory Osteolysis

**Figure 2:**
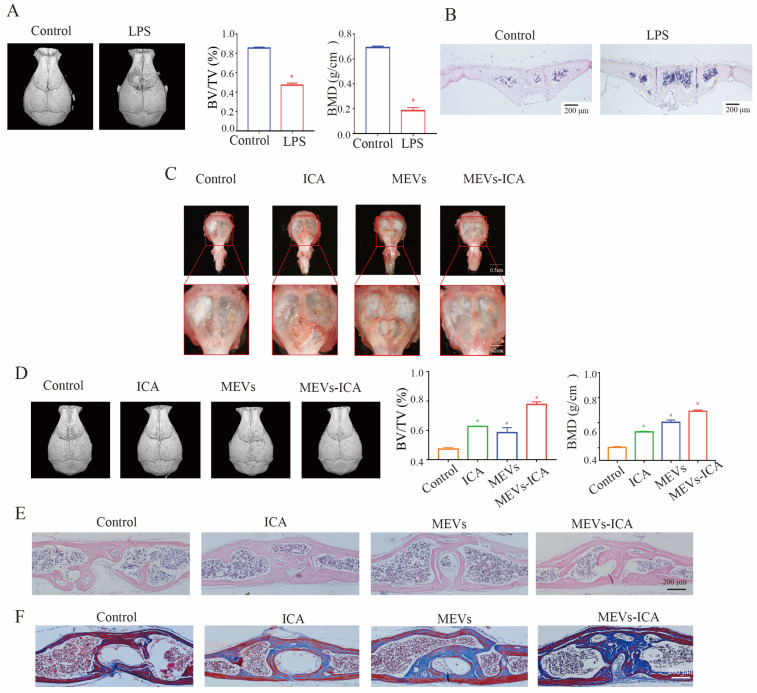
ICA-MEVs improve bone repair in LPS-induced inflammatory cranial defect mice. (**A**) Micro-CT showed rough bone surfaces and severe resorption in the LPS group (scale bar = 0.2 cm). (**B**) HE staining revealed exacerbated bone damage and thinned cortical bone in LPS lesions (scale bar = 200 μm). (**C**) Macroscopic observation of cranial defect morphology (scale bars = 0.5 cm, 0.2 cm). (**D**) Representative micro-CT images of cranial defects (scale bar = 0.2 cm). (**E**) HE staining of skull defect tissues (scale bar = 100 μm). (**F**) Masson staining evaluated collagen deposition during bone repair (scale bar = 100 μm). * *p* < 0.05 vs. control group.

Micro-CT three-dimensional reconstruction and bone morphometric analysis demonstrated that mice in the LPS group exhibited rough skull surfaces, a significant increase in bone resorption lacunae, and significantly lower bone volume fraction (BV/TV) and bone mineral density (BMD) compared with those in the control group (*p* < 0.05, Figure 2A). HE staining revealed expanded bone destruction areas, thinning of the cortical bone, and widening of the medullary cavity in the LPS group, accompanied by extensive infiltration by osteoclasts and inflammatory cells. These findings confirmed the successful establishment of an LPS-induced mouse model of skull inflammatory osteolysis (Figure 2B).

Following intervention, compared with the PBS group, mice in the ICA, MEV, and ICA-MEV groups showed varying degrees of improvement in skull gross morphology and bone structure, with the ICA-MEV group exhibiting the most prominent therapeutic effect (Figure 2C). Micro-CT findings indicated that the ICA-MEV group had the least bone resorption lacunae, with the skull surface basically restored to smoothness; additionally, the BV/TV and BMD in the ICA-MEV group were significantly higher than those in the other groups (*p* < 0.05) (Figure 2D). HE and Masson staining showed that the ICA-MEV group had the smallest osteolysis area, significantly restored cortical bone continuity, remarkably reduced infiltration of osteoclasts and inflammatory cells, and abundant formation of new bone tissue and collagen fibers (Figure 2E,F).

**Figure 3:**
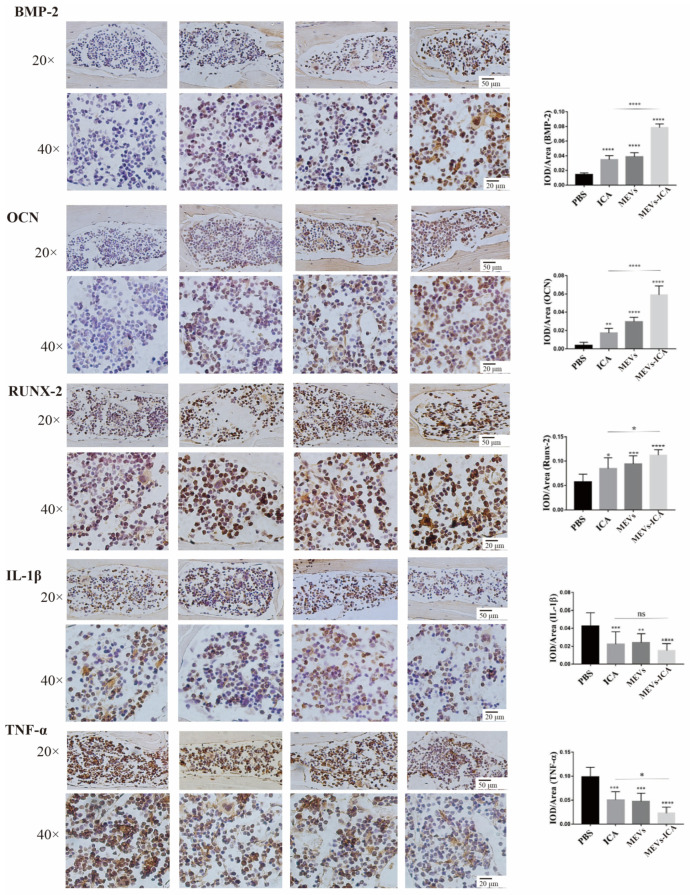
Expression of osteogenic factors (BMP-2, OCN, Runx-2) and inflammatory factors (IL-1β, TNF-α) in mouse skull defects. Scale bars = 50 μm and 20 μm. ns: no statistically significant difference; * *p* < 0.05, ** *p* < 0.01, *** *p* < 0.001, **** *p* < 0.0001 (vs. the corresponding control group).

Immunohistochemical staining at 20× magnification was performed to locate and quantify target protein expression in cranial defect tissues. Quantitative analysis demonstrated that ICA-MEV treatment markedly elevated the levels of osteogenic markers (BMP-2, OCN, Runx-2) and significantly reduced the expression of pro-inflammatory mediators (IL-1β, TNF-α), with all differences exhibiting extreme statistical significance (*p* < 0.0001). These findings indicated that ICA-MEVs alleviate LPS-triggered inflammatory osteolysis, facilitate osteogenic matrix formation, and attenuate inflammatory infiltration and bone resorption (Figure 3).

### 3.3. ICA-MEVs Promoted Proliferation and Differentiation of MC3T3-E1 Cells in an LPS-Mediated Inflammatory Microenvironment

**Figure 4:**
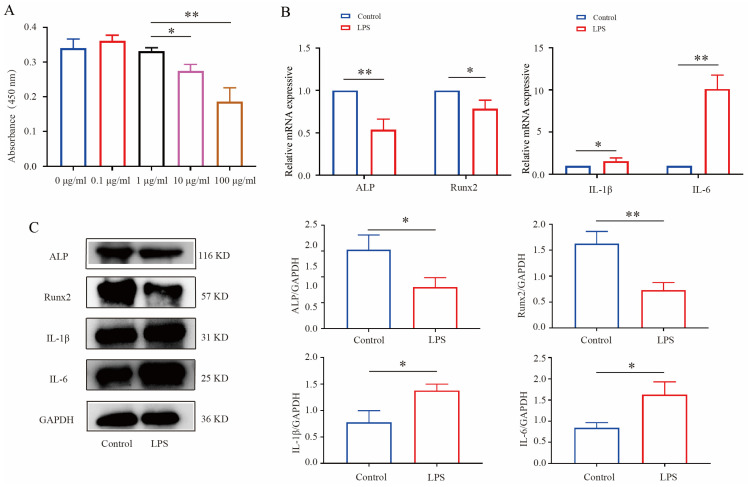
LPS suppresses osteogenic gene expression and enhances inflammatory responses in MC3T3-E1 cells. (**A**) CCK-8 results verified that 10 and 100 μg/mL LPS markedly inhibited cell viability and triggered cytotoxicity in MC3T3-E1 cells. (**B**) qRT-PCR detection confirmed that LPS treatment downregulated the mRNA levels of osteogenic ALP and RUNX2, whereas it upregulated the pro-inflammatory IL-1β and IL-6 transcripts. (**C**) Western blot assays further validated the consistent protein expression trends of the above osteogenic and inflammatory genes. * *p* < 0.05, ** *p* < 0.01 versus the control group.

**Figure 5:**
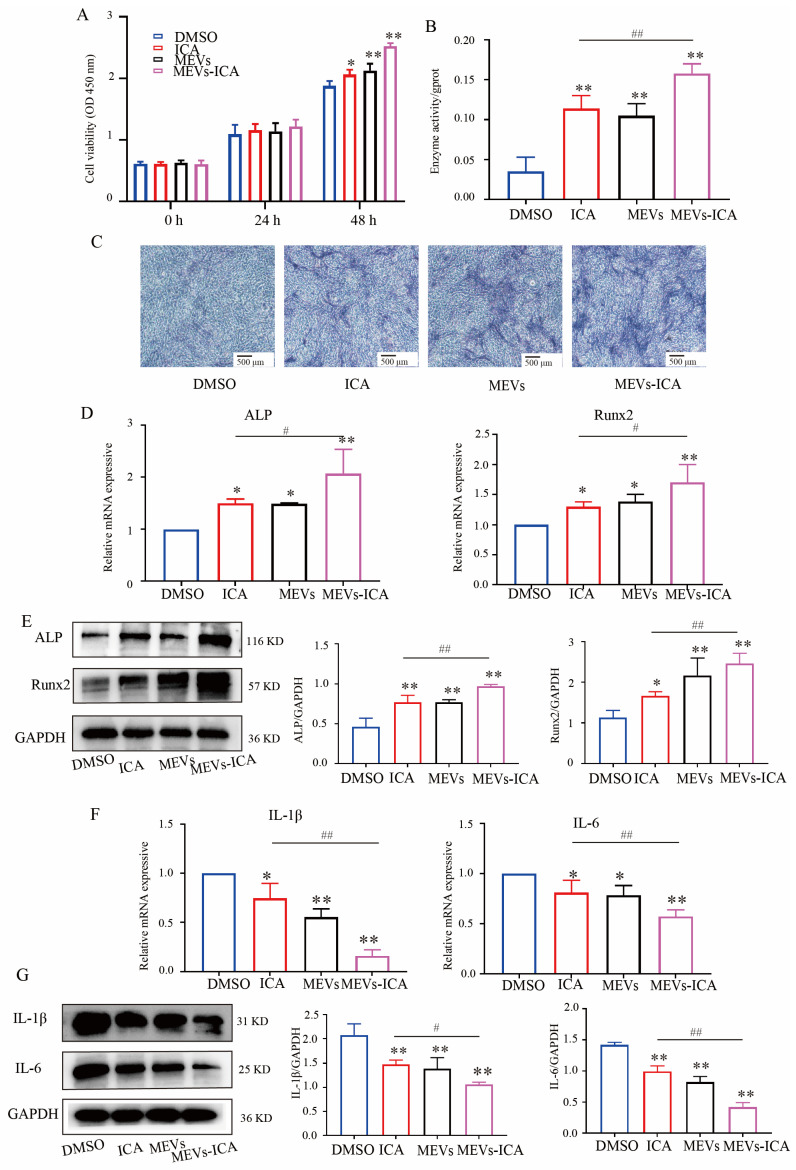
ICA-MEVs regulate proliferation, osteogenic differentiation and inflammation in LPS-stimulated MC3T3-E1 cells. (**A**) CCK-8 assays detected ICA-MEVs’ regulatory effect on the proliferation of inflamed MC3T3-E1 cells. (**B**) ALP activity assay evaluated cellular osteogenic capacity. (**C**) ALP staining visualized osteogenic differentiation (scale bar = 500 μm). (**D**,**E**) qRT-PCR and Western blot examined mRNA and protein levels of osteogenic ALP and Runx2 under inflammation. (**F**,**G**) The two assays also detected the expression of inflammatory IL-1β and IL-6. * *p* < 0.05, ** *p* < 0.01 vs. control group; ^#^ *p* < 0.05, ^##^ *p* < 0.01 vs. ICA group.

MC3T3-E1 cells were treated with 0–100 μg/mL LPS for 24 h. CCK-8 assays showed that 10 and 100 μg/mL LPS significantly suppressed cell proliferation and induced cytotoxicity (*p* < 0.05), so 1 μg/mL LPS was selected for subsequent modeling (Figure 4A). This concentration downregulated ALP and Runx2 while upregulating IL-1β and IL-6 (*p* < 0.05; Figure 4B,C), indicating that LPS inhibited osteogenic differentiation and promoted inflammation. In the 1 μg/mL LPS-induced inflammatory model, ICA and ICA-MEVs both enhanced cell proliferation, with ICA-MEVs being more effective (*p* < 0.05; Figure 5A). After 7 days of osteogenic induction, ICA-MEVs showed a stronger differentiation-promoting effect than ICA (*p* < 0.01; Figure 5B,C). Forty-eight hours post-intervention, real-time PCR and Western blotting confirmed that ICA-MEVs significantly increased ALP and Runx2 expression (*p* < 0.05; Figure 5D,E) and exerted the strongest inhibition on IL-1β and IL-6 (*p* < 0.05; Figure 5F,G). Collectively, ICA-MEVs promoted MC3T3-E1 cell proliferation and osteogenic differentiation and reduced pro-inflammatory cytokines under inflammatory conditions, with superior efficacy compared to ICA alone.

### 3.4. Transcriptomic Analysis of ICA-MEV Regulation of MC3T3-E1 Cell Proliferation and Differentiation Under Inflammatory Conditions

**Figure 6:**
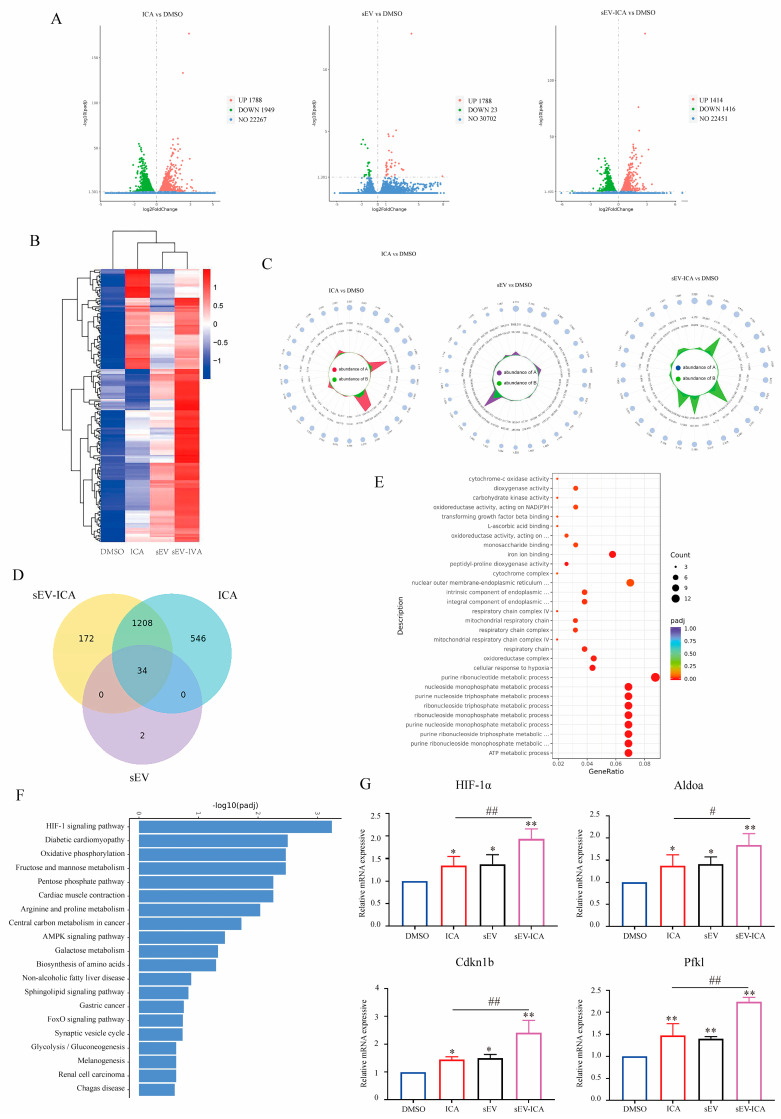
Transcriptome screening identified HIF-1α as a core differentially expressed gene in ICA-MEV-treated MC3T3-E1 cells. (**A**) Volcano plots showing global DEG distribution after ICA-MEV stimulation. (**B**) Heatmap illustrating DEG expression clustering in the treatment group. (**C**) Radar plots depicting core DEG expression profiles. (**D**) Venn diagrams comparing enriched DEG numbers among groups. (**E**) Top ten GO terms covering molecular function, cellular component and biological process. (**F**) KEGG analysis showing significantly enriched pathways of screened DEGs. (**G**) qRT-PCR validation of HIF-1α, Aldoa, Cdkn1b and Pfkl mRNA levels. * *p* < 0.05, ** *p* < 0.01 vs. control; ^#^ *p* < 0.05, ^##^ *p* < 0.01 vs. ICA group.

Compared with the DMSO group, the ICA, MEV, and ICA-MEV groups displayed distinct differentially expressed genes (DEGs). Volcano plot analysis identified 1788 up- and 1949 down-regulated genes in the ICA group, 36 up- and 23 down-regulated genes in the MEV group, and 1414 up- and 1416 down-regulated genes in the ICA-MEV group (Figure 6A). Hierarchical clustering and radar plots visualized the expression profiles of up-regulated DEGs and the top 30 core up-regulated genes (Figure 6B,C). Venn diagram analysis revealed 172 unique DEGs specifically regulated by ICA-MEVs (Figure 6D).

Gene Ontology (GO) and Kyoto Encyclopedia of Genes and Genomes (KEGG) functional enrichment analyses were performed on 172 screened differential genes. The results illustrated that these DEGs were primarily annotated to molecular function, cellular component and biological process categories (Figure 6E), and the HIF-1 signaling pathway was identified as a key pathway mediating the therapeutic effects of ICA-MEVs (Figure 6F). Four significantly altered genes in the HIF-1 pathway, including Hif-1α, Aldoa, Cdkn1b and Pfkl, were further selected for qRT-PCR verification. Compared with the DMSO control group, the expression of these four genes was distinctly elevated in the ICA, MEV and ICA-MEV groups, with the ICA-MEV group showing remarkably higher gene levels than the pure ICA group (*p* < 0.05). Among them, Hif-1α presented stable and the most significant expression changes. As a key transcription factor responsible for the anti-inflammatory and pro-osteogenic functions of milk-derived ICA-MEVs, HIF-1α was ultimately determined as the core target for subsequent mechanistic investigation (Figure 6G).

### 3.5. Effects of ICA-MEVs on the Interaction Between LIM1 and the HIF-1α Promoter Under Inflammatory Conditions

**Figure 7:**
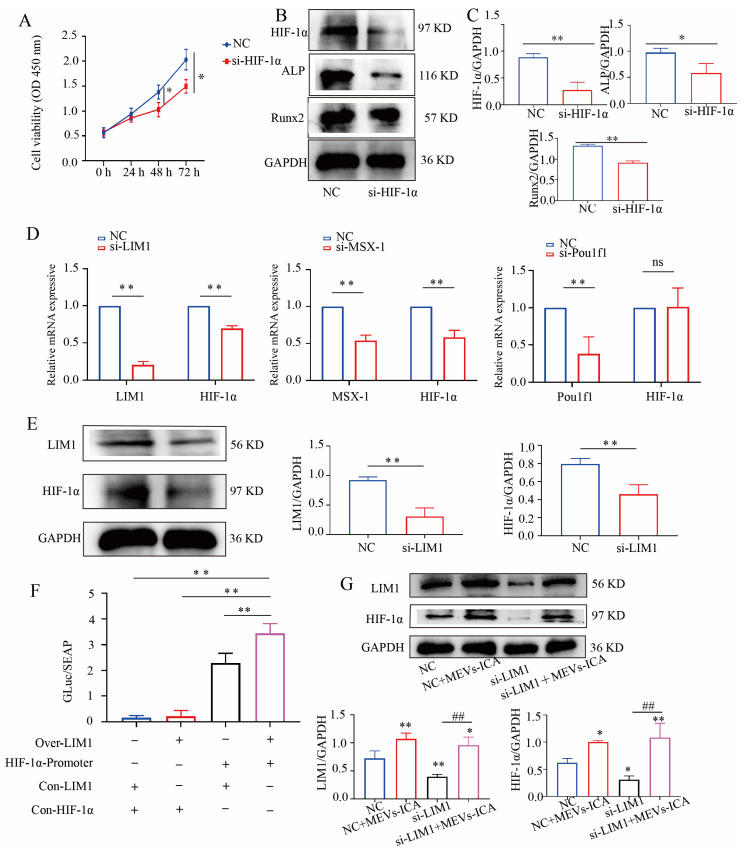
Mechanistic verification of the LIM1/HIF-1α axis under inflammation. (**A**) CCK-8 assays evaluated the proliferation of inflamed MC3T3-E1 cells after HIF-1α knockdown. (**B**) Western blot detected osteogenic ALP and Runx2 expression following HIF-1α downregulation. (**C**) Quantitative analysis of HIF-1α, ALP and Runx2 protein levels. * *p* < 0.05, ** *p* < 0.01 vs. control. (**D**) qRT-PCR examined HIF-1α transcriptional changes after LIM1, MSX-1 and Pou1f1 silencing. (**E**) Western blot confirmed altered HIF-1α expression upon LIM1 knockdown. (**F**) Luciferase assays tested HIF-1α promoter activity following Over-LIM1 co-transfection under inflammation. (**G**) Western blot assessed LIM1 and HIF-1α protein expression. ns, no significant difference; * *p* < 0.05, ** *p* < 0.01 vs. control; ^##^ *p* < 0.01 vs. ICA group.

Based on the above results, stably expressed HIF-1α was selected as the key target for further investigation. Functional rescue experiments showed that HIF-1α knockdown significantly suppressed MC3T3-E1 cell proliferation (CCK-8, *p* < 0.01) (Figure 7A) and reduced expression of the osteogenic markers ALP and Runx2 (Western blot, *p* < 0.05) (Figure 7B,C) under LPS stimulation. Collectively, these results demonstrate that HIF-1α contributes to the regulation of MC3T3-E1 cell proliferation and osteogenic differentiation in the inflammatory microenvironment.

To explore the regulatory mechanism by which ICA-MEVs modulated HIF-1α under inflammatory conditions, the PROMO (Profiler of Multi-Omic data) database and literature-based prediction were used to identify candidate transcription factors. LIM1, MSX-1, and Pou1f1 were predicted to potentially bind to the HIF-1α promoter and activate its transcription. Real-time PCR demonstrated that, under inflammatory conditions, knockdown of LIM1 and MSX-1 markedly reduced HIF-1α mRNA expression (*p* < 0.01), whereas silencing of Pou1f1 exerted no significant effect on HIF-1α expression (*p* > 0.05) (Figure 7D). Given its relatively higher transfection efficiency, LIM1 was selected for subsequent Western blot analysis. Downregulation of LIM1 decreased HIF-1α protein expression (*p* < 0.01) (Figure 7E), preliminarily verifying that LIM1 activates HIF-1α transcription.

A HIF-1α promoter luciferase reporter plasmid (HIF-1α-Promoter) and a LIM1 overexpression plasmid (Over-LIM1) were constructed and co-transfected into cells. Luciferase activity assays revealed that the co-transfection group exhibited the highest luciferase activity (*p* < 0.01) (Figure 7F), confirming that LIM1 binded to the HIF-1α promoter and promoted its transcription under inflammatory conditions. An inflammatory cell model was established by stimulating MC3T3-E1 cells with 1 μg/mL LPS for 24 h. Cells were then subjected to LIM1 knockdown with or without ICA-MEV treatment. Western blot results showed that ICA-MEVs alone significantly elevated the protein levels of both LIM1 and HIF-1α (*p* < 0.05); knockdown of LIM1 decreased HIF-1α expression (*p* < 0.05); and combined LIM1 knockdown plus ICA-MEV treatment significantly restored the expression of both proteins (*p* < 0.01) (Figure 7G). Collectively, these data demonstrated that under inflammatory microenvironments, ICA-loaded MEVs upregulate HIF-1α in a LIM1-dependent manner, which further facilitates the proliferation and osteogenic differentiation of MC3T3-E1 osteoblasts.

## 4. Discussion

Clinical inflammatory bone repair materials, including gelatin sponges, hydroxyapatite, bone grafts, and nanocollagen, were limited by low osteogenic efficiency and inadequate biodegradability [8,9,10]. These shortcomings often led to delayed wound healing and secondary infection, constraining their clinical translation [11,12]. In recent years, bioactive components from traditional Chinese medicine have attracted increasing attention in bone regeneration research [13]. As a typical isoflavone, ICA has been verified to mitigate inflammatory bone destruction by enhancing osteoblast proliferation and differentiation, suppressing excessive inflammation, and inhibiting osteoclast activity [14,15]. However, the clinical application of ICA is severely hampered by its intrinsic drawbacks, including poor water solubility and low bioavailability.

To address these limitations, extensive studies have focused on constructing ICA-loaded composite systems. For example, ICA-loaded porous β-tricalcium phosphate (β-TCP) ceramics promoted the proliferation and differentiation of rat osteoblasts in vitro and induced ectopic bone formation in vivo [16]. Similarly, ICA immobilization on titanium implants achieved sustained drug release and improved osteoblast adhesion and differentiation [17,18]. In our previous work, we successfully developed ICA-MEVs and verified their superior osteogenic activity compared with free ICA [19]. Nevertheless, the effects and underlying molecular mechanisms of ICA-MEVs on MC3T3-E1 cell proliferation and osteogenic differentiation under inflammatory conditions remained unclear. The present study was therefore designed to investigate the osteogenic potential and molecular basis of ICA-MEVs in an inflammatory microenvironment.

We conducted free drug removal experiments via repeated ultracentrifugation and size-exclusion chromatography. After thorough washing, the detectable ICA content remained stable, which excludes the possibility that ICA only loosely adheres to the MEV surface. Second, multiple published studies on small molecule-loaded milk EVs have confirmed that passive incubation loading predominantly encapsulates hydrophobic small molecules inside the vesicle lumen rather than surface adsorption [20,21,22,23]. Direct visualization (e.g., fluorescence co-localization microscopy) will be implemented in our follow-up research to directly trace ICA distribution inside MEVs.

Functional assays demonstrated that ICA-MEVs significantly enhanced the viability of MC3T3-E1 cells, as shown by CCK-8 assay, with a more pronounced effect than ICA. Histochemical staining and quantitative analysis revealed markedly elevated activity of ALP, an early marker of osteogenic differentiation, in the ICA-MEV group. Consistent with these results, Western blotting and RT-qPCR confirmed that ICA-MEVs upregulated the gene and protein expression of ALP and the key osteogenic transcription factor Runx2. Meanwhile, ICA-MEVs effectively reduced expression of the pro-inflammatory cytokines IL-1β and IL-6, exerting a stronger anti-inflammatory effect than the other treatments. These results indicated that ICA-MEVs synergistically promoted proliferation and osteogenic differentiation of MC3T3-E1 cells in an inflammatory environment. Accumulating studies have demonstrated the critical roles of TRAP activity, macrophage polarization and the RANKL/OPG axis in inflammatory bone loss. Combined with our osteogenic phenotypes, we speculated that ICA-MEVs suppresses osteoclastogenesis via balancing the RANKL/OPG ratio and reducing TRAP-positive osteoclasts. Meanwhile, ICA-MEVs induced M2 macrophage polarization to mitigate inflammatory response, thereby simultaneously restraining bone resorption and facilitating bone regeneration.

The LPS-induced mouse calvarial osteolysis model used herein represents chronic inflammatory bone loss, as local LPS stimulation was sustained for 14 days. Unlike acute short-term LPS exposure (≤72 h), which elicits transient self-limited inflammation, prolonged 14-day endotoxin challenge creates a persistent pro-inflammatory niche, with sustained pro-osteoclastogenic cytokine release and progressive cumulative bone erosion. This model recapitulated key pathological characteristics of clinical chronic inflammatory bone disorders driven by persistent microbial endotoxin stimuli, such as chronic apical periodontitis and peri-implantitis, where sustained low-grade inflammation leads to gradual, irreversible focal bone resorption in patients.

To elucidate the underlying molecular mechanism, transcriptomic profiling and KEGG pathway analysis were performed. The results revealed a close association between the osteogenic activity of ICA-MEVs and the HIF-1 signaling pathway, with significantly upregulated HIF-1α expression. HIF-1α is critically involved in skeletal homeostasis and bone disease progression by modulating osteoclast and osteoblast differentiation. Previous studies reported that HIF-1α stability is essential for osteogenesis, and HIF-1α knockdown blunts the osteogenic effect of pharmacological agents [24,25,26]. In line with these observations, our RT-qPCR verification confirmed that ICA-MEVs markedly increased HIF-1α expression in MC3T3-E1 cells. Moreover, HIF-1α downregulation significantly attenuated cell proliferation and osteogenic differentiation, confirming the central role of HIF-1α in ICA-MEV-mediated osteogenesis under inflammatory conditions.

Further exploration of the upstream regulators of HIF-1α identified the transcription factors LIM1, MSX-1, and Pou1f1 as candidate mediators. Knockdown of LIM1 and MSX-1 significantly reduced HIF-1α expression, whereas Pou1f1 silencing showed no significant effect, suggesting that LIM1 and MSX-1 positively regulated HIF-1α transcription. Among these, LIM1 was selected for mechanistic validation due to its stable transfection efficiency. LIM1 (LHX1), a member of the LIM homeodomain transcription factor family, participates in organ development and plasticity by forming transcriptional regulatory complexes [27,28]. However, its involvement in osteogenic differentiation and HIF-1α regulation has not been reported.

In vitro dual-luciferase reporter analysis demonstrated that LIM1 could bind and transcriptionally activate the HIF-1α promoter in exogenous cellular contexts, and maximal reporter activity was detected following simultaneous overexpression of LIM1 and the HIF-1α promoter construct. Functional rescue experiments showed that ICA-MEV intervention effectively reversed the HIF-1α downregulation induced by LIM1 deficiency. Collectively, these data suggested that ICA-MEVs elevated HIF-1α expression through LIM1-dependent modulation, which further promoted the proliferative capacity and osteogenic differentiation of MC3T3-E1 cells under inflammatory conditions. Of note, luciferase reporter assays alone could not fully validate the endogenous interaction and physiological occupancy of LIM1 on the HIF-1α promoter, leaving the in situ regulatory mechanism of this axis incompletely characterized. To address this technical limitation, future work will integrate ChIP assays with comprehensive molecular validation approaches to delineate the endogenous binding profile of LIM1 to HIF-1α. Such investigations will further consolidate the molecular mechanism underlying the LIM1/HIF-1α signaling cascade triggered by ICA-MEVs during osteogenic regulation. In summary, the present study identified a novel LIM1/HIF-1α regulatory cascade that mediated the osteogenic and anti-inflammatory bioactivities of ICA-MEVs. These findings offered substantial mechanistic evidence to support the prospective application of ICA-MEVs in the therapeutic repair of inflammatory bone defects.

Although ICA-MEVs possess prominent osteogenic and anti-inflammatory properties, their clinical transformation is currently hindered by challenges in scalable extracellular vesicle isolation, standardized icariin loading techniques, and the absence of unified global regulatory specifications. Traditional ultracentrifugation is unsuitable for large-scale industrial production, while the integration of tangential flow filtration and size-exclusion chromatography provides an alternative strategy for high-throughput and high-purity EV purification. The loading efficacy of icariin is highly dependent on incubation and electroporation parameters, which necessitates standardized operating procedures and consistent evaluation indicators to reduce batch-to-batch variability. Given the lack of universal regulatory criteria for food-derived extracellular vesicles, rigorous raw material quality control, as well as systematic batch safety and immunogenicity evaluation, are essential prior to clinical investigation. Continuous optimization of purification and drug-loading protocols, together with comprehensive biosafety validation, will greatly promote the clinical translational progress of ICA-MEVs.

The large-scale manufacturing of engineered MEVs still confronts multiple technical and standardization barriers. Classical ultracentrifugation protocols cannot meet the stringent requirements of clinical-grade production. In contrast, the combined application of SEC and tangential flow filtration effectively enhances the purification yield and structural purity of MEVs. Additionally, cryoprotectant usage and repeated freeze–thaw operations may compromise vesicle structural integrity and the biological activity of encapsulated cargos. Fluctuations in milk raw material sources and modification efficiency inevitably result in batch heterogeneity, highlighting the necessity of establishing unified quality release standards. All manufacturing procedures must strictly adhere to GMP guidelines, MISEV2023 criteria, and official pharmaceutical regulatory regulations [29,30]. Collectively, engineered MEVs represent a promising biomaterial candidate for oral mucosal rehabilitation, bone regenerative therapy, and the treatment of inflammatory disorders.

## Data Availability

The data presented in this study are contained within the article. Further inquiries can be directed to the corresponding authors.
